# Comparative Review of Environmental Audit Tools for Public Open Spaces from the Perspective of Children’s Activity

**DOI:** 10.3390/ijerph192013514

**Published:** 2022-10-19

**Authors:** Xue Meng, Mohan Wang

**Affiliations:** 1 School of Architecture, Harbin Institute of Technology, Harbin 150001, China; 2 Key Laboratory of Cold Region Urban and Rural Human Settlement Environment Science and Technology, Ministry of Industry and Information Technology, Harbin 150001, China; 3 School of Architecture, Harbin Institute of Technology (Shenzhen), Shenzhen 518055, China

**Keywords:** children, built environment, parks, play, physical activity, child-friendly environment

## Abstract

Public open spaces are important venues for children’s participation in outdoor activities and social life. This study performs a comparative and qualitative review of the tools that can be used to audit the environments of children-focused public open spaces. The analysis reviews 25 studies involving 11 tools for comparison. The results reveal that (1) the tools were developed in different fields; (2) the tools use two data resources, field investigation and geographic databases; (3) the tool dimensions are diverse, as are the number of items covered, and are generally related to four categories: surrounding environment and accessibility, activity and perceived safety, children’s sports and play opportunities, and aesthetic and comfort of the environment; (4) the reliability of most tools has been verified, with some validity still to be confirmed; (5) there are differences in tool users, settings, and aims. Among the tools, the CPAT and the EAPRS are the most comprehensive. Comparative analysis of the tools provides a reference for studies on children-focused public open spaces and for the development and improvement of corresponding tools in the future.

## 1. Introduction

Public open spaces are vital as venues for leisure and recreational activities, social interaction, and sports and fitness activities, among others. Thus, they are particularly important as spatial supports for social and cultural life in cities. Studies have shown that the use of public open spaces by residents, especially children, can significantly impact their physical and mental health [1,2,3,4,5]. Moreover, attractive public open spaces can effectively encourage children to participate more often in physical and play activities as well as promote their ability to interact with peers while playing a positive role in boosting attention, language, cognition, and general capabilities [6,7,8,9,10]. Moreover, general, public spaces are particularly valuable for children with disabilities and autistic disorders [11,12]. In addition, since the outbreak of COVID-19, many child-oriented facilities, such as schools and museums, have been closed for various periods, limiting the options for child-focused activities to some extent [13,14]. As such, the importance of public open spaces for children’s outdoor play, social interaction, and contact with nature has increased dramatically.

Evaluations of public open space environments through quantitative analyses of their availability and environmental quality can be valuable references for improving solutions to existing space environment-related problems. Such evaluations can also provide an in-depth understanding of the impact of environmental mechanisms on specific users and assist in optimal urban resource allocation planning. 

Over the past several decades, scholars have researched and systematically evaluated various types of public open spaces, including green spaces in neighborhoods and urban parks. At the same time, environmental audit tools have been developed in the fields of public health, urban and environmental design, and environmental psychology, among others. These include the Public Open Space Tool (POST), the Bedimo-Rung Assessment Tool–Direct Observation (BRAT-DO), and the Environmental Assessment of Public Recreation Spaces (EAPRS) [15,16,17]. Overall, this research stream has become more detailed and focused, with various scholars considering the characteristics of children and conducting relevant environmental assessments from this perspective. 

Researchers have also analyzed and reviewed audit tools related to neighborhood physical environments. Nickelson and colleagues reviewed the audit tools used to study the domains and subdomains of neighborhood physical environments [18]. Kan and colleagues [19] focused on audit tools measuring neighborhood built environments from the perspective of aging in place. Other scholars have reviewed neighborhood environmental audit tools from the perspective of walking and cycling [20,21,22]. In terms of open public spaces and parks, Joseph and Maddock analyzed five observational tools used to evaluate the suitability of park environments for physical activity, two of which focused on children’s needs [23]. However, no study has investigated park audit tools from the perspective of children’s usage.

Our study is the first to focus exclusively on audit tools that can assess a park’s physical environment from the perspective of children’s activities. Our objective is to summarize and describe the studies that cover park audit tools for children-focused public open spaces. We describe the basic information and characteristics of the tools, and compare them from the perspectives of field and development goals, data sources and tool forms, dimensions and items, public open space users, tool users, settings, and audit results. Our findings can help provide a basis for child-related environmental behavior research, help improve relevant tools, and support the improvement of public open space quality and the allocation of urban public space resources.

## 2. Literature Review Process

**Figure 1:**
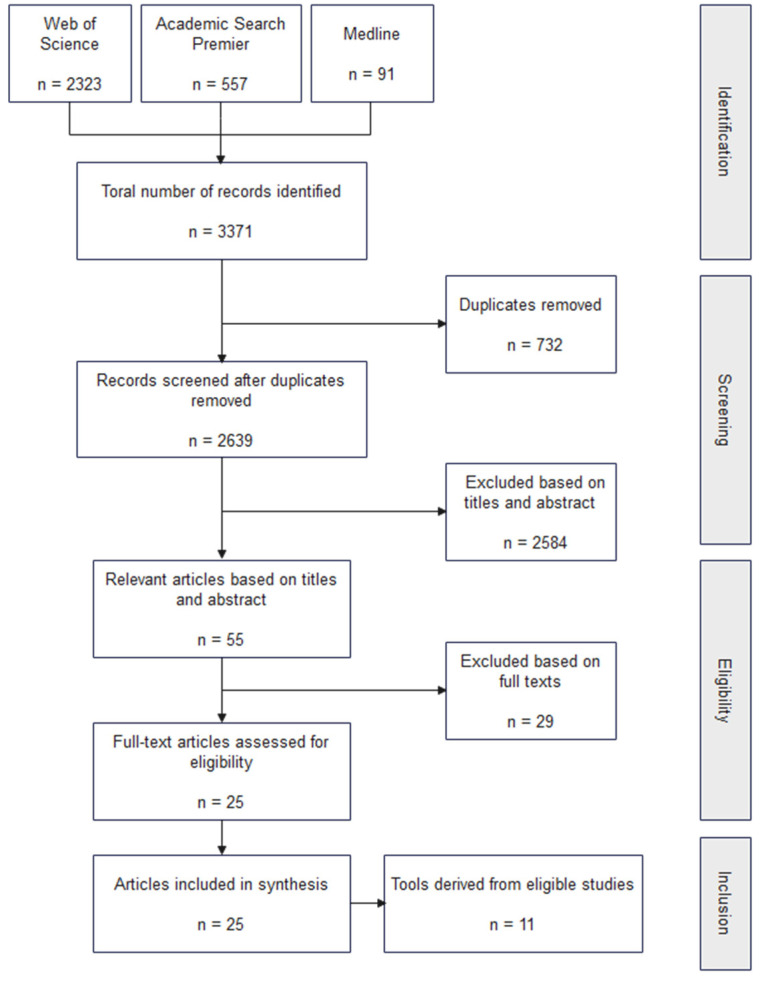
Flowchart of the literature review process.

In our literature review, we systematically investigated studies that focused on assessing the environment of child-focused public open spaces, with the aim of summarizing and categorizing the environmental audit tools described. We conducted our review in June 2022. We used the Web of Science, MEDLINE, and Academic Search Premier databases to identify the appropriate studies and searched using the following keywords in three categories: (1) audit tool-related—audit or instrument or assessment or scale or tool; (2) children-related—child* or toddler or adolescent or teen* or youth* or kid; and (3) space type-related—park or open space or recreation area or green space or play* space. The US-based site Active Living Research (https://activelivingresearch.org/) (accessed on 20 June 2022) was also used to compare public open space-related environmental audit tools to ensure no tool was overlooked. The search was not limited by publication date. Our review process is described in Figure 1. 

We then screened our search results for studies that met the following criteria: (1) published in a peer-reviewed journal; (2) described children (people under the age of 18) as the main users of the space; (3) included public open spaces in general; (4) focused on the physical environment of the space; (5) included an evaluation of the comprehensive quality of the public space; and (6) had the full text available in English.

Articles were excluded if they: (1) were non-research articles or not primary literature; (2) were not peer-reviewed articles, such as conference articles or academic dissertations; (3) focused on specific types of facilities (e.g., gymnasiums, child-care centers); (4) only evaluated a single dimensional property of public open spaces (e.g., safety); (5) did not mention detailed information on audit tools (e.g., purpose, dimensions, items); or (6) the full-text version was not available or not written in English. In addition, if a study used multiple audit tools but did not propose a new tool, we included it in the corresponding literature on the original tools (e.g., [24,25]). Ultimately, we included 25 studies that met our criteria, and from these we identified 11 unique audit tools for analysis.

## 3. Overview of Audit Tools and Comparison Analysis

### 3.1. Audit Tools

These studies included both the introduction of the given tools’ development and the tools’ applications. The tools identified were: the Environmental Assessment of Public Recreation Spaces (EAPRS) [17,26,27,28,29]; the Children’s Public Open Space Tool (C-POST) [30,31]; the Community Park Audit Tool (CPAT) [32,33,34,35,36,37,38]; the Physical Activity Resource Assessment (PARA, adapted version) [39]; the Resilience for Eating and Physical Activity Despite Inequality (READI) park audit tool [40]; the Woolley and Lowe’s play space assessment tool [41,42]; the Parks, Activity and Recreation among Kids (PARK) tool [43,44,45]; the Playable Space Quality Assessment Tool (PSQAT) [46]; the QUality INdex of Parks for Youth (QUINPY) [47]; the Opportunities for Children in Urban Spaces (OCUS) [48]; and the Play Space Audit Tool (PSAT) [49]. Among these, the EAPRS tool does not primarily target children but does include detailed index items and has been used or adapted in multiple studies evaluating children’s playgrounds. Thus, we included it in our comparative analysis.

We present an overview of these 11 tools in Table 1. All the tools were developed over the past 15 years (2006–2020), a relatively concentrated length of time. The EAPRS and the POST-based C-POST were developed relatively early, while the OCUS and the PSAT are more recent. Most of the tools have been used in national or regional projects targeting residents’ physical activity, dietary behavior, or health levels; a few studies do not detail the sources of their tools. The tools assess between 3 and 16 dimensional properties of public open spaces and cover between 22 and 744 individual items. All the tools originate in developed countries in the West, specifically, six in the United States, two in Australia, and one each in the United Kingdom, Canada, and Italy. The United States and Australia were the first to focus on the internal connection between public open spaces and public health. The POST, the BRAT-DO, the EAPRS, and the PARA tools were developed in these countries to evaluate the attributes of public space environments related to users’ physical activity. Subsequently, as the research stream deepened, children-focused audit tools were developed and published. Moreover, due to widespread attention on children as a vital target group in recent years, evaluating public space environments from a children’s activity perspective has become an important direction in public space environmental evaluation. Most of the tools have been developed independently from one another. Overall, we found that different countries and teams were conducting research in parallel while drawing on one another during their research on audit tools.

#### 3.1.1. Environmental Assessment of Public Recreation Spaces

The EAPRS tool is aimed at comprehensively measuring the physical environment of public recreational spaces, such as parks and play spaces [17]. Of the 11 tools, it is the most comprehensive and detailed, comprising 16 categories, including paths, water areas, facilities, and play structures, among others. The sixth revision covers 744 individual items targeting children’s activities, including over 200 in the play sets and sports structure category alone [50]. Many scholars have used the items in the tool to observe, evaluate, and empirically investigate children’s recreational spaces. Researchers have also shortened the tool to improve its flexibility and practicality [29].

#### 3.1.2. Children’s Public Open Space Tool

The C-POST is aimed at effectively evaluating the attributes of public open spaces, including parks, playgrounds, and green spaces, which can influence children’s physical activity [30]. It originated as the Public Open Space Tool (POST) [15] proposed by Broomhall and Giles-Corti, and was then simplified and improved to create the C-POST tool by making it more relevant to children. It includes three category dimensions that cover 27 assessment items. The categories are recreational facilities, availability of amenities, and other characteristics related to the environmental quality.

#### 3.1.3. Community Park Audit Tool

The CPAT [32] evaluates the quality of community park environments from a physical activity perspective. Results reflect the level of support a community park can provide for the users’ physical activity, with a focus on the park’s child-related use characteristics. The tool includes three dimensions that cover 118 assessment items. Although the tool includes a relatively high number of items, most of these can be responded to quickly with an objective judgment of “yes/no” or “present/absent.” Thus, the perceived quality of the community park can be assessed in a relatively short time. Another research team developed the eCPAT, an electronic version of the tool, which is a mobile application that offers a youth-friendly alternative to the traditional pencil-and-paper tool [37,38].

#### 3.1.4. Physical Activity Resource Assessment (Adapted Version)

The PARA audit tool aims to evaluate the physical activity resources available in a given neighborhood. It evaluates parks, playgrounds, sports facilities, fitness centers, and other places where residents can engage in physical activities [51]. It involves three dimensions—the features of physical activity resources, amenities, and incivilities (e.g., graffiti, litter, vandalism)—covering a total of 34 items. Subsequently, DeBate et al. proposed an adapted version of the tool focusing on children [39]. The item classifications were not changed, but one item each from amenities and incivilities was deleted in the adapted version, for a total of 32 items.

#### 3.1.5. READI Park Audit Tool

The READI tool is developed based on a study in response to obesity among groups of disadvantaged women and children [40]. It is aimed at identifying and assessing park features associated with the physical activity of park users (children and adults) and comparing environmental differences between urban and rural parks [40]. The tool has 11 category dimensions covering 84 items. Seven categories are child-related, including accessibility, lighting and safety, aesthetics, amenities, and playgrounds.

#### 3.1.6. Woolley and Lowe’s Play Space Assessment Tool

The audit tool proposed by Woolley and Lowe evaluates the level of support a space offers for children’s play [41]. Specifically, it evaluates outdoor activity spaces especially designed and designated as children’s play areas. Woolley and Lowe [41] have used the tool to evaluate 10 play areas in the English Midlands and validated the research assumption that “a more natural play space provides increased play value.” The tool is based on three dimensions: play type, physical elements, and environmental characteristics of the space, covering 22 items.

#### 3.1.7. Parks, Activity and Recreation among Kids

The PARK tool evaluates park features related to children’s physical and recreational activity [43]. This tool draws on and adapts the POST [15] and the BRAT-DO [16], two existing audit tools, adjusting the language of the assessment items and the measurement scale and adding 13 new items for a total of 92. The evolved version explores five dimensions: activities, environmental quality, services, safety, and general impression.

#### 3.1.8. Playable Space Quality Assessment Tool

The PSQAT aims to evaluate the quality of children’s play space considering social–environmental characteristics and factors related to the value of play [46]. It is appropriate for assessing children’s play spaces and activity facilities. It includes three dimensions covering a total of 24 items. The dimensions are location, play value, and care and maintenance. Additionally, PSQAT classifies play spaces into three types according to the size and scale of the park and its distance from the child’s home: door-step, which refers to a park within view of children’s homes; local; and neighborhood, which refers to larger facilities.

#### 3.1.9. Quality Index of Parks for Youth

The QUINPY is aimed at evaluating park quality in urban park systems from the perspective of park users’ needs and park usage [47], specifically focusing on the inclusiveness of parks for young people of different ages. This tool was also developed on the important premise that parks and recreation agencies in many large and medium-sized cities in the United States are continuously collecting basic park information in the process of improving investment decisions, thus providing basic statistical support for evaluations. The QUINPY has five dimensional categories covering 18 items. The dimensions are recreational diversity, natural environment, size, maintenance, and safety [47]. The tool uses publicly available geospatial data to evaluate the parks. Therefore, on-site audits are not required. The significant advantage of this tool is that it saves a considerable amount of time and funds. It also demonstrates a data-driven approach to park environment quality assessment.

#### 3.1.10. Opportunities for Children in Urban Spaces

The OCUS tool evaluates children’s experience in public open spaces. It was designed to evaluate the environmental characteristics of public open spaces considering affordability [48]. It includes four dimensions that cover a total of 30 items. The dimensions include: functional, social, emotional/contextual, and independent accessibility opportunities. The items cover both micro- and macro-scale environmental properties.

#### 3.1.11. Play Space Audit Tool

The PSAT tool quickly and effectively assesses the playability of a children’s playground [49]. It includes four dimensions: general playground overview; playground surface; pathways; and play equipment, covering 47 items. The developers tested the tool with 70 playground samples and verified its reliability.

### 3.2. Comparison of Audit Tools

As discussed, these audit tools vary in terms of their priorities and scope; thus, each one is more or less applicable in a given setting or scenario. In the following, we present a comparative analysis of the 11 tools (Table 2).

#### 3.2.1. Field and Development Purpose

Most of the 11 tools (n = 8) originated in the field of public health and are focused on assessing public open space environmental characteristics that can influence children’s physical activity in the context of health issues such as physical inactivity, obesity, and cardiovascular disease. Among these, the QUINPY tool draws on the results of public health and environmental psychology to evaluate park quality from the perspective of children’s needs and their park utilization [47]. Woolley and Lowe’s play space assessment tool [41], the PSQAT [46], and the PSAT [52] start from the perspective of environmental play value for children. They are based mainly on environmental psychology and landscape design and assess environmental support in public open spaces for children’s play. In general, differences in the field development and the aims of the tools can explain the differences in the dimensions and items covered.

#### 3.2.2. Data Sources and Tool Form

All the tools except the QUINPY require that evaluations be made based on field observations or pictures taken on-site. These field tools are implemented in pencil-and-paper form, with the assessors recording their evaluations manually according to their observations. The QUINPY relies on a publicly available database to obtain information about park environments for evaluation, and information unavailable in the database can be supplemented through aerial photos [47]. The tool is also notable in that scores for each item are computer-calculated, allowing the city parks to be ranked in an urban park system. The CPAT tool is available in an electronic format in the eCPAT app, allowing park users to conduct assessments on their mobile devices (e.g., cell phones and tablets) [37,38]. The OCUS tool combines on-site surveys and the geographic database available, but the form of the tool is not clearly stated [48].

#### 3.2.3. Dimensions and Items

The dimensions of the audit tools are summarized in Table 3. Of the 11 tools, nine have from three to five dimensions, which, in general, include recreational facilities, amenities, and characteristics related to environmental quality. Notably, the READI park audit tool and the EAPRS contain additional dimensions, 11 and 16, respectively. The dimensions in these two tools are also more detailed. For instance, referring to the recreational facilities, some sub-dimensions (e.g., paths, outdoor sports venues, play areas) are treated independently, thus creating additional dimensions.

**Figure 2:**
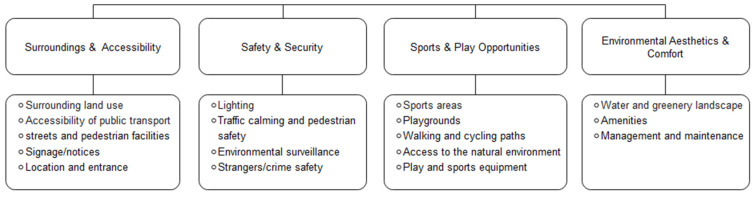
Audit tool dimensions and items.

The items the tools cover can be summarized in four categories: surroundings and accessibility, safety and security, children’s sports and play opportunities, and environmental aesthetics and comfort (Figure 2). The surroundings and accessibility category is intended to capture the likelihood that potential users will visit a public open space; the other three categories address the environmental quality of the public open space. Table 4 shows which items from each category are covered by each tool.

In terms of the scope of the items, the EAPRS, the CPAT, the READI park audit tool, the PARK, and the PSAT cover all four categories, and the items under each category are relatively comprehensive, especially in the CPAT and the EAPRS, the most comprehensive tools. The other tools focus mainly on the latter two categories. Among these, Woolley and Lowe’s play space assessment tool is the most limited in terms of items because it mainly targets the evaluation of children’s play without significant attention to other aspects.

#### 3.2.4. Users of Public Open Spaces

Of the 11 tools, eight have been specifically developed for children, two give special focus to children (i.e., CPAT and READI park audit tool), and one (i.e., EAPRS) does not limit participant groups. The tools target children in the following age ranges: (1) 8–12 years old—the stage characterized by high frequent physical activity during primary school; (2) 5–12 years old—primary school stage; (3) 5–18 years old—primary school and adolescent stage; and (4) children of all age groups. About half of the tools emphasize primary school children’s activities. Although the EAPRS tool does not target specific users, it places considerable importance on the opinions and attitudes of the parents of young children who frequently use parks being considered during the research and development process of park planning, which implies an emphasis on children.

#### 3.2.5. Users of the Tools

Most of the tools (except for OCUS, which is not reported) are suitable for use by researchers. The CPAT and the PARA (adapted version) are also suitable for community residents, community managers, and other non-professionals because the items are easy to understand. The evaluation method is also sufficiently simple that rapid assessment can be realized through assessors’ objective reporting of environmental conditions alone. The eCPAT, the electronic version of the CPAT, is also suitable for use by young people. Generally, the other tools are used only by professional researchers due to the high literacy and experience requirements. Some tools require users to participate in training sessions to ensure a better understanding of the tools and higher evaluation accuracy.

#### 3.2.6. Environmental Setting

In terms of setting, the EAPRS, the C-POST, the PARA (adapted version), and the OCUS apply to parks, play venues, green spaces, and other public open spaces. Among these, the PARA (adapted version) focuses on a physical activity resource evaluation. As such, it is not limited to types of public open spaces but can be applied to stadiums, schools, and other facilities that support physical activity. The CPAT, the PARK, the READI park audit tool, and the QUINPY primarily target parks. Woolley and Lowe’s play space audit tool, the PSAT, and the PSQAT are suitable for evaluating children’s play areas. The former two focus on the quality of specifically designated children’s play spaces, while the latter applies to formal children’s play spaces and informal activity spaces (such as open areas near home).

#### 3.2.7. Auditing Results

Of the 11 tools, seven (EAPRS, C-POST, CPAT, READI park audit tool, PARK, PSQAT, PSAT) report the reliability of their auditing results. The results reveal moderate to high inter-rater reliability. Some tools (C-POST, READI park audit tool, PARK) also show intra-rater reliability. Reliability does not apply to the QUINPY, as the QUINPY is a secondary data-based tool. Since the original version of the PARA confirmed its reliability, we can infer that the adapted version of the PARA also has acceptable reliability [51]. In general, this indicates the reliability of most tools is verified, while the tool proposed by Woolley and Lowe and the OCUS still require verification. Only one tool (QUINPY) confirmed its validity, as the researchers compared it with rankings by experts. Thus, validity still needs to be confirmed for the other tools.

Regarding the application of the audits, most of the tools can be used for auditing public open spaces. Thus, the results of such audits can reflect specific environmental characteristics and be used for environmental behavior research (such as environmental impacts on children’s physical activity). Studies that compare contexts (such as different socio-economic regions) or the environmental equity of open spaces could also be conducted. The QUINPY tool is unique in that it ranks parks within a city’s park system according to their quality scores. Therefore, it is unsuitable for use in evaluating individual parks independently. The audit results can help create a better understanding of the overall quality of park systems within a city and provide reliable support for improvement and maintenance. It can also be helpful for the allocation of public space resources on a larger scale.

## 4. Discussion

After over a decade of development, scholars from various countries have made significant progress in the environmental assessment of public open spaces, focusing substantial attention on children. According to our comparative study, the 11 audit tools vary in terms of development field and motivation, data source, dimensions, and items covered. Additionally, they can be applied to different needs and scenarios.

The aims of these tools can be summarized as follows. The first is to measure how well different public open spaces support children’s physical activity or play to provide feedback that can guide future design and practice. The second is to validate the key material and environmental factors that affect children’s physical activity and play behavior. This can provide a scientific basis for improving the environment of activity spaces by integrating behavioral observation methods (e.g., SOPARC, SOPLAY, behavior mapping) or behavior recording tools (e.g., GPS, accelerator). The third is to compare public open space environments within different cities/regions and neighborhoods with different social and economic conditions and find solutions by drawing more attention to the improvement of social equality and spatial equity among groups of disadvantaged children. Furthermore, some studies combine one or more audit tools to assess the equity of public open space distribution and its impact in cities or wider areas, considering both quality and quantity [46,52].

In line with the development and improvement of relevant research, we note the following trends among the audit tools. First, data acquisition methods are becoming increasingly diversified, developing from traditional field research and observation techniques to technical approaches, such as geographic information and remote sensing image collection [47,52,53]. Second, the scope of research has gradually expanded from the evaluation of general spaces for children’s activities (such as public open spaces and parks) in the initial stage to the evaluation of specific places such as primary and secondary schools [54]. Third, the research is increasing in depth as it extends beyond the overall evaluation of environmental quality to detailed observations of a particular dimension (such as safety) [55]. Fourth, multiple audit tools have been used simultaneously within one study to obtain environmental characteristics at different scales and conduct a more comprehensive study (e.g., obesogenic environmental assessment, spatial equity) [36,56].

The effectiveness and reliability of all the audit tools have been validated as they have been widely applied and promoted. However, some problems have also been exposed, for example, the fairness of the evaluation results. As the scale of the evaluation object has an important impact on the potential for the allocation of various resources, such as sports and play areas, equipment, and landscapes, larger-scale public open spaces (or parks, play areas) tend to score higher than small- and medium-sized spaces [17,41]. In such cases, the results cannot truly and objectively reflect the quality of the environment and the level of support that a given park provides for children’s activities. Additionally, most tools are limited to use in specific regions, and their effectiveness for wider areas has not been tested. As such, there is still considerable space for further research and regional adaptation of the tools. Moreover, other detailed points for children should be considered when developing future tools. For instance, the distance between the playground and the parking lot can affect children’s respiratory health [57]. Thus, items related to spatial connection or separation should also be considered.

## 5. Conclusions

Our study is the first to present a literature-based overview of 11 public open space audit tools that focus on children’s activity and then compare their characteristics. Our results indicate that the existing tools for auditing public open spaces focus considerable attention on children and have made numerous breakthroughs in recent years in terms of scope and accuracy owing to in-depth study and the integration of new technologies. However, the results on support for children’s activity in public spaces are not comparable due to different starting points and perspectives among the projects. In the future, relevant tools should be expanded and developed based on previous work while comprehensively considering how children will use and experience the space. Furthermore, new data sources (such as Google Street View and remote sensing images) should be incorporated to increase the efficiency and decrease the costs of public space assessment. Doing so could also enhance the applicability of these audit tools, thereby providing a basis for their promotion, comparison, and validation in different regions and across a wider scope.

The importance of outdoor public spaces for children’s activity cannot be more evident. Reconsidering the environmental quality of urban public spaces from the perspective of children’s activity and conducting relevant scientific assessments remain significant endeavors.

## Figures and Tables

**Table 1:** Summary of the audit tools.

No.	Audit Tool	Authors	Year	No. of Dimensions	No. of Items	Country	References
1	EAPRS	Saelens et al.	2006	16	744	US	[17,26,27,28,29]
2	C-POST	Crawford et al.	2008	3	27	Australia	[30,31]
3	CPAT	Kaczynski et al.	2010	3	118	US	[32,33,34,35,36,37,38]
4	PARA (adapted version)	DeBate et al.	2011	3	32	US	[39]
5	READI park audit tool	Veitch et al.	2012	11	84	Australia	[40]
6	Woolley and Lowe’s play space assessment tool	Woolley & Lowe	2013	3	22	UK	[41,42]
7	PARK	Bird et al.	2015	5	92	Canada	[43,44,45]
8	PSQAT	Jenkins et al.	2015	3	24	US	[46]
9	QUINPY	Rigolon and Nemeth	2016	5	18	US	[47]
10	OCUS	Garau and Annunziata	2019	4	30	Italy	[48]
11	PSAT	Gustat et al.	2020	4	47	US	[49]

Note: The authors and year the tools were originally reported.

**Table 2:** Audit Tool Characteristics.

Tool	Field	Main Aim	Study Subjects (Age Group)	Data Collection	Setting	Tool Users	Reliability	Validity
EAPRS	Public health	To assess the quality of the physical environment in public recreational spaces	General users	Field study	Parks, playgrounds, green spaces	Researchers	Some 65% to 69% of the items have a good–excellent range or high percent agreement	Not reported
C-POST	Public health	To assess the features of public open spaces related to children’s physical activity	Children (5 to 12 years old)	Field study	Parks, playgrounds, green spaces	Researchers	All items have at least adequate intra- and inter-rater reliability	Not reported
CPAT	Public health	To assess community park characteristics related to children’s physical activity	Children (unspecified age) and general adults	Field study	Community parks	Researchers, community residents, community stakeholders	90% of the items have good to excellent (>70%) agreement Of the items where kappa coefficients (n = 84) could be calculated, 78.6% have moderate to high agreement (k > 0.40)	Not reported
PARA (adapted version)	Public health	To assess the features of children’s physical activity resources within neighborhoods	Children (8 to 12 years)	Field study	Various physical activity resources	Researchers, community residents, community stakeholders	Not reported	Not reported
READI park audit tool	Public health	To assess park characteristics related to users’ physical activity	Children (5 to 12 years old) and general adults	Field study	Parks	Researchers	Intra-rater reliability for each domain ranges from 70% to 100% agreement (mean percent agreement) Inter-rater reliability for each domain ranges from 81% to 100% agreement (mean percent agreement)	Not reported
Woolley and Lowe’s play space assessment tool	Environmental psychology, landscape and environmental design	To evaluate the environmental characteristics and play value of children’s playgrounds	Children (all ages)	Field study	Playgrounds	Researchers	Not reported	Not reported
PARK	Public health	To assess park characteristics related to children’s physical activity and recreational activity	Children and teenagers (5 to 18 years old)	Field study	Parks	Researchers	Some 86% of items have good to excellent (≥75%) agreement Of the items where kappa coefficients (n = 79) could be calculated, 85% have moderate to high agreement (k > 0.40) Most items (i.e., all but 7) have moderate to high intra-rater reliability (k > 0.40)	Not reported
PSQAT	Environmental psychology, landscape and environmental design	To evaluate the environmental features of public open spaces considering play value and social environment	Children (all ages)	Field study	Play spaces, playgrounds	Researchers	Inter-rater reliability for each domain is excellent (ICC > 0.85). Internal consistency reliability for each domain is good to excellent (95% CI = 0.76 to 0.97, 0.53 to 0.95, 0.53 to 0.95)	Not reported
QUINPY	Public health, environmental psychology	To assess the environmental quality of urban parks for children’s use	Children (all ages)	Geographic databases	Parks	Researchers, practice designers	Not Applicable	Excellent (compared to experts’ rankings)
OCUS	Environmental psychology	To measure and evaluate the affordances of open spaces for children’s use	Children (all ages)	Field study and geographic databases	Public open spaces	Not reported	Not reported	Not reported
PSAT	Public health	To assess the environmental features of playgrounds for children’s play	Children (all ages)	Field study	playgrounds	Researchers	Most domains (all but “general playground overview,” which was not reported) have good to high inter-rater reliability (k ≥ 0.79)	Not reported

Note: Reliability and validity estimates are reported according to the original publication of each tool.

**Table 3:** Summary of tool dimensions.

Audit Tool	No. of Dimensions	Dimension Names	References
EAPRS	16	Trails; Paths; General areas; Water areas; Eating/drinking features; Facilities; Educational/historical features; Sitting or resting features (non-trail); Landscaping; General aesthetics; Access-related features; Directives and information-related features; Safety-related features; Play set or structure features; Other play components (not part of play set); Athletic fields and other recreation areas	[50]
C-POST	3	Recreational facilities; Availability of amenities; Other characteristics related to environmental quality	[30]
CPAT	3	Access and surrounding neighborhood; Park activity areas; Park quality and safety	[32]
PARA (adapted version)	3	Resources for physical activity; Amenities; Incivilities	[39]
READI park audit tool	11	Access; Lighting/safety; Aesthetics; Amenities; Playgrounds; Diversity of playground equipment; Safety/condition of playground equipment; Age appropriateness of playground equipment; Paths; Outdoor courts/sports ovals; Informal play spaces	[40]
Woolley and Lowe’s play space assessment tool	3	Play types; Physical elements of the space; Environmental characteristics of the space	[41]
PARK	5	Activities; Environmental quality; Services; Safety; General impression	[43]
PSQAT	3	Location; Play value; Care and maintenance	[46]
QUINPY	5	Structured play diversity; Nature; Park size; Maintenance; Safety	[47]
OCUS	4	Functional opportunities; Social opportunities; Emotional/contextual opportunities; Independent accessibility opportunities	[48]
PSAT	4	General playground overview; Surface, terrain, and vegetation; Pathways; Play structure and equipment	[49]

**Table 4:** The relationship between audit tool items and four key categories.

Audit Tool	Surroundings and Access					Safety and Security				Sports and Play Opportunities					Environmental Aesthetics and Comfort		
	Surrounding Land Use	Accessibility of Public Transport	Streets and Pedestrian Facilities	Signage/Notices	Location and Entrance	Lighting	Traffic Calming and Pedestrian Safety	Environmental Surveillance	Strangers/ Crime Safety	Sports’ Areas	Playgrounds	Walking and Cycling Paths	Access to the Natural Environment	Play and Sports Equipment	Water and Greenery Landscape	Amenities	Management and Maintenance
EAPRS	○		○	○	○	○	○	○	○	○	○	○	○	○	○	○	○
C-POST						○				○		○			○	○	
CPAT	○	○	○	○	○	○	○	○	○	○	○	○	○	○	○	○	○
PARA (Adapted Version)					○	○				○		○		○	○	○	○
READI park audit tool		○	○		○	○	○	○		○	○	○		○	○	○	○
Woolley and Lowe’s play space assessment tool											○		○	○	○		
PARK		○	○			○	○	○		○	○	○			○	○	○
PSQAT					○			○		○	○		○	○		○	○
QUINPY									○	○	○	○	○		○	○	○
OCUS		○				○	○	○			○	○		○	○	○	○
PSAT				○		○	○				○	○	○	○	○	○	○

## Data Availability

Data sharing is not applicable to this article.
